# Estimation of the Gini coefficient for the lognormal distribution of income using the Lorenz curve

**DOI:** 10.1186/s40064-016-2868-z

**Published:** 2016-07-28

**Authors:** Kwasi A. Darkwah, Ezekiel N. N. Nortey, Anani Lotsi

**Affiliations:** Department of Statistics, School of Physical and Mathematical Sciences, College of Basic and Applied Sciences, University of Ghana, Accra, Ghana

**Keywords:** Lorenz curve, Gini coefficient, Newton–Cotes methods, Lognormal distribution, Income distribution

## Abstract

The main objective of the study is to compare the Newton–Cotes methods such as the Trapezium rule, Simpson 1/3 rule and Simpson 3/8 rule to estimate the area under the Lorenz curve and Gini coefficient of income using polynomial function with degree 5. Comparing the Gini coefficients of income computed from the Polynomial function with degree 5 for the Trapezium, Simpson 1/3 and Simpson 3/8 methods using the relative errors showed that the trapezium rule, Simpson’s 1/3 rule and Simpson’s 3/8 rule show negative biases with the Simpson 1/3 rule yielding the lowest absolute relative true error of 4.230711 %.

## Background

The concept of inequality is broad as compared to poverty. This is because the definition of inequality is based on the whole population and not only a section of the population below a definite poverty line. Several measures of inequality must satisfy six basic criteria to become an accepted measure of inequality. They are; statistical testability, mean independence, Pigou–Dalton transfer sensitivity, population size independence, symmetry and decomposability (Glewwe 1986).

The simple inequality measure of a population separates those who are poor from those who are rich and shows the percentage of income ascribed to every quintile or decile of the population. The quintile which shows those who are poor are mostly 6–10 % of all income and the highest quintile fall within 35–50 %. Gini (1912) and Bassmann et al. (1990) have all devised various ways of measuring income inequality. The importance of measuring income on national basis is to ensure an efficient management of the financial and economic policies that will ensure price stability and economic growth.

According to Edozien (1991), poor health, illiteracy, unemployment and poverty are some characteristics of people who receive low income while literacy, stable jobs, sufficient health care are the characteristics of high income earners.

The size of income inequality is mostly measured and interpreted by the Lorenz curve. The Lorenz curve is a graph that accounts for the cumulative percentage of household income to the cumulative percentage of income received when the households are put in ascending order of their income.

Gini coefficient is one of the most efficient and commonly used measurement of income inequality in the world. It is computed as twice the region between a Lorenz curve and the egalitarian line. Gini coefficient as a measure of inequality is widely used in various contexts such as energy, credit availability, income, health care and wealth (Berndt et al. 2003).

This indeed confirms Morgan (1962) statement that the Gini index is the best single measure of inequality. The Gini coefficient is analyzed based on discrete and continuous distributions (Yitzhaki and Schechtman 2005). Golden (2008) demonstrated how estimates of Gini coefficient can be computed using numerical integration method.

Fellman (2012) estimated the Gini coefficient of income by employing numerical integration methods such as the Trapezium rule and Simpson’s rule. His research revealed that the Trapezium rule yield positive biases for the Lorenz curve while the Gini coefficient yielded negative biases.

Darkwah et al. (2016a) recently used numerical integration methods such as Boole and Weddle methods to compare the Gini coefficient of Ghanaian income from the 2013 Ghana Living Standard Survey (GLSS6) which were estimated using Rasche et al. (1980) function and polynomial function. Their results revealed that the estimated area under the Lorenz curve and Gini coefficients using Rasche et al. (1980) function and polynomial function according to the Boole and Weddle method of integration resulted in positive and negative biases respectively but the Weddle’s method was better as compared to Boole method of numerical integration in estimating the Gini coefficient of income with the Boole method producing the highest absolute relative error.

The main objective of the study is to compare the Newton–Cotes methods such as the Trapezium rule, Simpson 1/3 rule and Simpson 3/8 rule to estimate the area under the Lorenz curve and Gini coefficient of income using polynomial function with degree 5.

## Methods

All data used in this study was based on secondary data from the Ghana Living Standards Survey (GLSS6). The data had a total income of GhC 244,759,213.2 from a total of 58,788 individuals.

Let the vector $z = (z_{1}, z_{2}, z_{3}, \ldots, z_{n} )\,$ denote the distribution of individual income in a population with $i = 1,2,3, \ldots ,n$ and *n* is the total number of individuals. The income distribution has mean and density function as $\mu (z)\,{\text{and}}\,f(z)$ respectively. The Lorenz curve and Gini index of income for the lognormal distribution is given as $\Phi \left( {\Phi ^{ - 1} (z) - \sigma } \right)$ and $2\Phi \left( {\sigma /\sqrt 2 } \right) - 1$ respectively where $\sigma$ is the standard deviation and $\Phi (z)$ is the standard normal distribution (Cowell 1995).

Suppose $\pi (z)$ shows the cumulative proportion of the individuals that receive income up to z and $\eta (z)$ shows the cumulative proportion of the total income that is received by individuals in the same population. The Lorenz curve shows the relationship that exists between $\pi \,{\text{and}}\,\eta$. The Lorenz curve is established by letting *z* be the income of every individual and $f(z)$ be the probability density function of $z$. Hence

1 $$\pi (z) = \int\limits_{0}^{z} {f(y)} dy$$

and

2 $$\eta (z) = \frac{1}{\mu }\int\limits_{0}^{z} {yf(y)dy}$$

The mean of the distribution of income is;

3 $$\mu = \int\limits_{0}^{\infty } {yf(y)dy}$$

Ryu and Slottje (1996) declared that a function is fitted to the Lorenz curve in other to estimate the area under the Lorenz curve. A polynomial function proposed by Becker and Weispfenning (1993) is chosen from the several functions suggested for estimating the area under the Lorenz curve. Becker and Weispfenning (1993) suggested a polynomial which is a mathematical expression involving a sum of powers in one or more variables multiplied by coefficients to estimate the area under the Lorenz curve. A univariate polynomial with constant coefficients is given by;

4 $$\eta (\pi ) = a_{m} \pi^{m} + a_{m - 1} \pi^{m - 1} + \cdots + a_{1} \pi + a_{0}$$

When $a_{m} \ne 0\;{\text{and}}\;m \ge 2,$ the polynomial function is a continuous non-linear function.

The area under the Lorenz curve was estimated using a polynomial function with degree 5.

The estimate of the Lorenz curve is given by

5 $$L(\pi ) = \int\limits_{0}^{1} {\eta (\pi )d\pi }$$

The Gini coefficient which measures the inequality among the distribution of a nation’s residential income is given by;

6 $${\text{Gini}} = \,1 - 2\int\limits_{0}^{1} {\eta (\pi )d\pi }$$

The random variable $Z$ is modelled using the lognormal distribution with probability density and cumulative distribution function of $f_{{(\mu ,\sigma^{2} )}} (z) = \frac{1}{{z\sqrt {2\pi \sigma^{2} } }}e^{{ - \frac{{(Inz - \mu )^{2} }}{{2\sigma^{2} }}}} ,\;z > 0,\,\;\sigma > 0,\,\;\mu > 0$ and $F(z) = \phi \left( {\frac{Inz - \mu }{\sigma }} \right)$ respectively, where $Inz$ is normally distributed. The mean of $Z$ is $E(z) = e^{{\mu + \sigma^{2} /2}}$ and the variance of $Z$ is $V(z) = \left( {e^{{\sigma^{2} }} - 1} \right)e^{{2\mu + \sigma^{2} }}$.

The estimated parameters of the Lognormal distribution function from the Ghana household data on income would be integrated using the Newton–Cotes method such as the Trapezium rule, Simpson’s 1/3 rule and Simpson 3/8 rule to compute the Gini coefficient. The Newton–Cotes method involves $n$ points in the interval $\left[ {a,b} \right]$ with $n - 1$ order polynomial which passes through points $z_{i}$ and are equally spaced. Approximating the area under the curve $y = f(z)$ from $z = a$ to $z = b$, using the Trapezium rule, Simpson 1/3 rule and Simpson 3/8 rule with $z_{0} = a,\,z_{n} = b\,{\text{and}}\,h = \frac{(b - a)}{n}$ is (Sauer 2012; Darkwah et al. 2016b; Mettle et al. 2016).

For Trapezoidal rule, we have;

7 $$A = \frac{h}{2}\left[ {f(z_{0} ) + f(z_{1} )} \right]$$

For Simpson 1/3 rule, we have;

8 $$A = \frac{h}{3}\left[ {f(z_{0} ) + 4f(z_{1} ) + f(z_{2} )} \right]$$

For Simpson 3/8 rule, we have;

9 $$A = \frac{3h}{8}\left[ {f(z_{0} ) + 3f(z_{1} ) + 3f(z_{2} ) + f(z_{3} )} \right]$$

The relative errors are used to compared the Trapezium rule, Simpson 1/3 rule and Simpson 3/8 rule employed to estimate the Gini coefficients of the various regions, rural and urban areas, male and female heads. The relative error is computed as;

10 $$\varepsilon_{a} = \frac{Absolute\,Errors}{Exact}$$

Where $Absolute\,Error = Exact - Approximate$ and the number of significant digits at least correct is given as;

11 $$m = 2 - \log \left( {\frac{{\left| {\varepsilon_{a} } \right|}}{0.02}} \right)$$

The bias is calculated as;

12 $$Bias = Approximate\, - \,Exact$$

The Romberg numerical integration was used to calculate the “exact” numerical integration method. A general expression for Romberg integration can be written as (Sauer 2012; Darkwah et al. 2016b);

13 $$I_{2n}^{(k)} = \frac{{4^{k} I_{2n}^{(k - 1)} - I_{n}^{(k - 1)} }}{{4^{k - 1} - 1}},\quad k \ge 2$$

The index k represents the order of extrapolation and $I_{n}^{(1)}$ represents the values obtained from the regular trapezoidal rule with n intervals. Using $k = 2$ which represents values obtained using the true estimate as $o(h^{2} ).$ Hence

14 $$I_{true} \cong \,I_{true,est} = I_{2n} + \frac{{I_{2n} - I_{n} }}{3}$$

The main objective of this study is to compare the Gini coefficients of income computed using the Trapezium rule, Simpson 1/3 rule and Simpson 3/8 rule.

## Results

To apply the methodology proposed by this study, data on gross income was taken from the sixth round of the Ghana Living Standard Survey (GLSS 6) conducted by the Ghana Statistical Service (GSS). The data comprises of a nationwide sample of 58,788 family size with a total income of GHC 244,759,213.2. The mean and standard deviation of the Ghanaian income are 14,788.18 and 46,257.611 respectively. With a maximum income of 2,184,471 and a minimum income of 2, a substantial difference exists between the maximum and minimum income values which is an indication of a very high disparity in the income levels of Ghanaians. Also, skewness of 16.709 and kurtosis of 479.105 show that the data deviates widely from the normal distribution. The Kolmogorov–Smirnov (KS) test was used to test whether the distribution of the income is lognormal. The null hypothesis of the data following a lognormal distribution, produced a p value of 0.42 greater than 0.05 which is consistent with the lognormal.

Consequently, the total income $(z)$ is modeled using the lognormal distribution with parameters $\mu \,{\text{and}}\,\sigma^{2}$. The density function of the income which follows the lognormal distribution is given by

15 $$f_{{(\mu ,\sigma^{2} )}} (z) = \frac{1}{{z\sqrt {2\pi \sigma^{2} } }}e^{{ - \frac{{(Inz - \mu )^{2} }}{{2\sigma^{2} }}}} ,\quad z > 0,\quad \sigma > 0,\quad \mu > 0$$

The maximum likelihood estimators of the parameters $\mu \,{\text{and}}\,\sigma^{2}$ of the lognormal distribution based on the income distribution are given as $\hat{\mu } = \frac{{\sum\nolimits_{i} {Inz_{i} } }}{n} = 8.5311\,$ and $\hat{\sigma }^{2} =\frac{{\sum\nolimits_{i} {(Inz_{i} - \hat{\mu })^{2} } }}{n} = 1. 45812.$

The cumulative distribution function of the income is also given by;

16 $$F(z) = \phi \left( {\frac{Inz - 8.5311}{1.45812}} \right)$$

The Kolmogorov–Smirnov test which is used to test whether the distribution of income for the various regions, rural and urban areas and family heads is lognormal are shown in Table 1. Also the estimated Gini coefficient of the lognormal distribution can also be found in Table 1.

**Table 1:** Kolmogorov–Smirnov test, estimates of the mean, standard deviation and Gini index for lognormal distribution of the Ghanaian income

Region	Kolmogorov–Smirnov test		Estimated mean	Estimated standard deviation	Gini index
	Statistic	p value			
Greater Accra	0.0313	0.18486	8.7613	1.2724	0.63174
Eastern	0.0241	0.2353	8.6814	1.3380	0.6559
Ashanti	0.021	0.3548	8.7915	1.3570	0.66272
Volta	0.0291	0.1964	8.5908	1.3640	0.6652
Western	0.0257	0.2471	9.0076	1.3462	0.65886
Brong Ahafo	0.0244	0.2733	8.5370	1.3702	0.6674
Central	0.0219	0.4305	8.1159	1.2877	0.63746
Northern	0.0217	0.4014	8.3369	1.3222	0.65018
Upper West	0.0342	0.06939	7.8074	1.3215	0.64992
Upper East	0.031	0.1381	8.1671	1.4132	0.68234
Rural	0.0276	0.2226	8.2519	1.2533	0.6245
Urban	0.0305	0.1102	7.2546	1.3411	0.65702
Male head	0.0271	0.2251	8.2114	1.3252	0.65128
Female head	0.0269	0.2462	8.2322	1.4021	0.67852
All	0.0221	0.4201	8.5311	1.45812	0.69748

From Table 1, all the p values of the Kolmogorov–Smirnov tests of the various regions, rural and urban areas, male and female family heads are greater than 0.05 which is consistent with the lognormal. Also from Table 1, the estimates of the Gini index for lognormal distribution was found to be between 0.63174 and 0.68234 with respect to the regions where Greater Accra and Upper East region recorded the lowest and highest income inequality measured using the Gini index for the lognormal distribution.

Given $\pi (z)$ to be the proportion of the units that receive income up to $z$ and $\eta (z)$ to represent the proportion of total income received by the same units whose income are less than or equal to *z*, then using the Polynomial function with degree 5 to compute the estimates of the area under the Lorenz curve for nonlinear income distribution, the estimate of the area under the Lorenz curve using the Trapezium rule, Simpson 1/3 rule and Simpson 3/8 rule are shown in Table 2.

**Table 2:** Estimates of the area under the Lorenz curve using Trapezium, Simpson 1/3 and Simpson 3/8 numerical integration methods

Region	Trapezium rule	Simpson’s 1/3 rule	Simpson’s 3/8 rule	Exact integration
Greater Accra	0.2764	0.2684	0.2704	0.2623
Eastern	0.2675	0.2593	0.2626	0.2523
Ashanti	0.2655	0.2599	0.2613	0.2517
Volta	0.2690	0.2622	0.2656	0.2562
Western	0.2428	0.2426	0.2427	0.2370
Brong Ahafo	0.2632	0.2593	0.2611	0.2165
Central	0.2633	0.2603	0.2612	0.2553
Northern	0.2379	0.2319	0.2328	0.2304
Upper East	0.2296	0.2196	0.2235	0.2095
Upper West	0.3126	0.2246	0.2278	0.2143
Rural	0.2566	0.2530	0.2533	0.2513
Urban	0.2754	0.24465	0.2475	0.2258
Male head	0.2702	0.2613	0.2619	0.26
Female head	0.2618	0.23525	0.2389	0.2171
All	0.2660	0.2483	0.2504	0.23855

The estimate of the Gini coefficient of income using the estimate of the area under the Lorenz curve from the Trapezium rule, Simpson 1/3 rule and Simpson 3/8 rule is shown in Table 3 below.

**Table 3:** Estimates of Gini coefficient using the Trapezium, Simpson 1/3 and Simpson 3/8 numerical integration methods

Region/family head	Trapezium rule	Simpson’s 1/3 rule	Simpson’s 3/8 rule	Exact integration
Greater Accra	0.4472	0.4632	0.4592	0.4754
Eastern	0.4650	0.4814	0.4748	0.4954
Ashanti	0.4690	0.4802	0.4774	0.4966
Volta	0.4620	0.4756	0.4688	0.4876
Western	0.5144	0.5148	0.5146	0.526
Brong Ahafo	0.4736	0.4814	0.4778	0.567
Central	0.4734	0.4794	0.4776	0.4894
Northern	0.5242	0.5362	0.5344	0.5392
Upper East	0.5408	0.5608	0.5530	0.581
Upper West	0.3748	0.5508	0.5444	0.5714
Rural	0.4868	0.4940	0.4934	0.4974
Urban	0.4492	0.5107	0.5050	0.5484
Male head	0.4596	0.4774	0.4762	0.48
Female head	0.4764	0.5295	0.5222	0.5658
All	0.4680	0.5034	0.4992	0.5229

The computation of the Biases and Relative errors using the Trapezium, Simpson 1/3 and Simpson 3/8 numerical integration method is shown in Table 4.

**Table 4:** Biases generated from the Gini coefficient using Trapezium, Simpson 1/3 and Simpson 3/8 numerical integration methods as compared with the exact estimation

Region/family head	Trapezium rule	Simpson’s 1/3 rule	Simpson’s 3/8 rule
Greater Accra	−0.0282	−0.0122	−0.0162
Eastern	−0.0304	−0.014	−0.0206
Ashanti	−0.0276	−0.0164	−0.0192
Volta	−0.0256	−0.012	−0.0188
Western	−0.0116	−0.0112	−0.0114
Brong Ahafo	−0.0934	−0.0856	−0.0892
Central	−0.016	−0.01	−0.0118
Northern	−0.015	−0.003	−0.0048
Upper East	−0.0402	−0.0202	−0.028
Upper West	−0.1966	−0.0206	−0.027
Rural	−0.0106	−0.0034	−0.004
Urban	−0.0992	−0.0377	−0.0434
Male head	−0.0204	−0.0026	−0.0038
Female head	−0.0894	−0.0363	−0.0436
All	−0.0549	−0.0195	−0.0237

From Table 5 below, the highest relative error of 34.40672 % was computed using the Trapezium numerical integration method for the Upper West Region while the lowest relative error of 0.55638 % was computed from the Simpson 1/3 numerical integration method for the Northern Region. Also, the highest relative error for the family heads was 15.80064 % for female family head computed from the Trapezium rule while the lowest relative error for the family heads was 0.541667 % for male family head computed from the Simpson 1/3 rule. The Urban area had the highest relative error of 18.08899 % from the Trapezium rule while the Simpson 1/3 rule was used to compute the lowest relative error of 0.683554 % for rural area.

**Table 5:** Relative Error generated from the Gini coefficient using Trapezium, Simpson 1/3 and Simpson 3/8 numerical integration methods as compared with the exact estimation

Region/family head	Trapezium	Rule	Simpson’s 1/3	Rule	Simpson’s 3/8	Rule
	Relative error (%)	m	Relative error (%)	m	Relative error (%)	m
Greater Accra	5.931847	2	2.566260	2	3.407657	2
Eastern	6.136455	1	2.825999	2	4.158256	2
Ashanti	5.557793	2	3.302457	2	3.866291	2
Volta	5.250205	2	2.461034	2	3.855619	2
Western	2.205323	2	2.129278	2	2.167300	2
Brong Ahafo	16.47266	1	15.09700	1	15.73192	1
Central	3.269309	2	2.043318	2	2.411116	2
Northern	2.781899	2	0.556380	2	0.890208	2
Upper East	6.919105	1	3.476764	2	4.819277	2
Upper West	34.40672	1	3.605180	2	4.725236	2
Rural	2.131082	2	0.683554	2	0.804182	2
Urban	18.08899	1	6.874544	1	7.913931	1
Male head	4.25000	2	0.541667	2	0.791667	2
Female head	15.80064	1	6.415695	1	7.705903	1
All	10.49914	1	3.729203	2	4.532415	2

## Conclusion

The main aim of the study was to compare the estimates of the Gini coefficient of income computed using the Trapezium rule, Simpson 1/3 rule and Simpson 3/8 rule. The results showed that positive biases were generated for the area under the Lorenz curve for all the Newton–Cotes methods such as the Trapezium rule, Simpson 1/3 rule and Simpson 3/8 rule used while from Table 4, the Gini coefficient generated negative biases. This shows that there is no uniform optimization for the Newton–Cotes methods used. From Table 5, the Trapezium method generated the highest relative error of 34.6583 % for the Upper West Region while the lowest relative error of 0.81391 % was computed from the Simpson 1/3 method for the Northern Region. Also, using the entire income data, the Trapezium rule yielded the highest relative error of 10.97626 % while the Simpson 1/3 rule yielded the lowest relative error of 4.230711 %. This shows that the Simpson’s 1/3 is the best method compared to Trapezium rule and Simpson 3/8 rule in estimating the Gini coefficient of income. This confirms Darkwah et al. (2016a) and Fellman (2012) findings that the area under the Lorenz curve and Gini coefficient yields negative and positive biases. Also, Trapezium rule, Simpson 1/3 rule and Simpson 3/8 rule applied to the Polynomial of order 5 gave lesser estimates of the Gini coefficient of income when compared to the estimates of the Gini index for the lognormal distribution in Table 1. Future research could use other Newton–Cotes methods such as Boole and Weddle applied to other Lorenz curve functions such as Gupta (1984) and Ortega et al. (1991) to estimate the Gini coefficient and compare them to the lognormal distribution estimates of the Gini coefficient. It is also recommended to investors, policy makers, financial analysts and stakeholders in the world that when choosing Newton–Cotes methods to compute the inequality of income of their countries, they should make use of the Simpson 1/3 numerical integration method instead of the Trapezium rule and Simpson 3/8 rule.
