# Erratum to: Estimation of the Gini coefficient for the lognormal distribution of income using the Lorenz curve

**DOI:** 10.1186/s40064-016-2990-y

**Published:** 2016-08-10

**Authors:** Kwasi A. Darkwah, Ezekiel N. N. Nortey, Anani Lotsi

**Affiliations:** Department of Statistics, School of Physical and Mathematical Sciences, College of Basic and Applied Sciences, University of Ghana, Accra, Ghana

## Erratum to: SpringerPlus (2016) 5:1196 DOI 10.1186/s40064-016-2868-z

Upon publication of the original article (Darkwah et al. 2016), it was noticed that Anani Lotsi’s name was accidentally spelt ‘Anane Lotsi’. This has now been corrected in the original article.
